# Competition structured a Late Cretaceous megaherbivorous dinosaur assemblage

**DOI:** 10.1038/s41598-019-51709-5

**Published:** 2019-10-28

**Authors:** Jordan C. Mallon

**Affiliations:** 1 Beaty Centre for Species Discovery and Palaeobiology Section, Canadian Museum of Nature, PO Box 3443, Station D, Ottawa, Ontario K1P 6P4 Canada; 2 Department of Earth Sciences, Carleton University, 2115 Herzberg Laboratories, 1125 Colonel By Drive, Ottawa, Ontario K1S 5B6 Canada

**Keywords:** Evolution, Palaeontology

## Abstract

Modern megaherbivore community richness is limited by bottom-up controls, such as resource limitation and resultant dietary competition. However, the extent to which these same controls impacted the richness of fossil megaherbivore communities is poorly understood. The present study investigates the matter with reference to the megaherbivorous dinosaur assemblage from the middle to upper Campanian Dinosaur Park Formation of Alberta, Canada. Using a meta-analysis of 21 ecomorphological variables measured across 14 genera, contemporaneous taxa are demonstrably well-separated in ecomorphospace at the family/subfamily level. Moreover, this pattern is persistent through the approximately 1.5 Myr timespan of the formation, despite continual species turnover, indicative of underlying structural principles imposed by long-term ecological competition. After considering the implications of ecomorphology for megaherbivorous dinosaur diet, it is concluded that competition structured comparable megaherbivorous dinosaur communities throughout the Late Cretaceous of western North America.

## Introduction

The question of which mechanisms regulate species coexistence is fundamental to understanding the evolution of biodiversity^1^. The standing diversity (richness) of extant megaherbivore (herbivores weighing ≥1,000 kg) communities appears to be mainly regulated by bottom-up controls^2–4^ as these animals are virtually invulnerable to top-down down processes (e.g., predation) when fully grown. Thus, while the young may occasionally succumb to predation, fully-grown African elephants (*Loxodonta africana*), rhinoceroses (*Ceratotherium simum* and *Diceros bicornis*), hippopotamuses (*Hippopotamus amphibius*), and giraffes (*Giraffa camelopardalis*) are rarely targeted by predators, and often show indifference to their presence in the wild^5^. Rather, food resources tend to be limiting to such large animals, particularly during the dry season when food is less abundant^2,3^.

**Figure 1:**
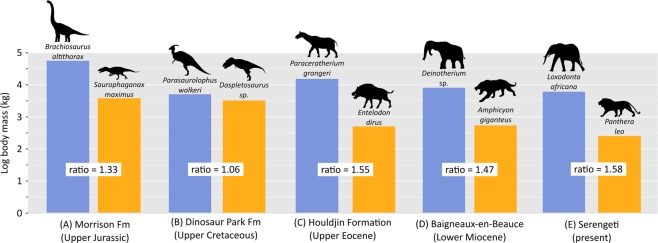
Ratios of log-transformed body mass of largest herbivore (in blue) to largest carnivore (in orange) for select fossil and modern ecosystems. Examples (A) and (B) illustrate dinosaur ecosystems; examples (C)–(E) illustrate mammal ecosystems. Note that herbivore:carnivore size ratios are closer to 1 in the dinosaur ecosystems; that is, predator and prey are more nearly equal in size. Silhouettes not to scale. Abbreviation: Fm, Formation.

It is axiomatic that our understanding of modern megaherbivore ecology is based entirely on the study of mammal communities, these being the only megaherbivores alive today^5^. However, for most of the Mesozoic, only dinosaurs occupied this category, and it is not obvious that megaherbivorous dinosaur communities were similarly immune to those same top-down processes that their living counterparts shirk today. Of particular note is the fact that dinosaurian predators were much larger than those of today^6^ (Fig. 1), and likely would have posed a significant threat to even the largest herbivores of their time (with the possible exception of the biggest sauropods). This is especially true if large theropods were capable of cooperative hunting, a hypothesis that has garnered some support from both bonebed and trackway evidence^7–9^. Large theropod bite marks have also been recorded on the bones of massive ceratopsids, hadrosaurids, sauropods, and stegosaurs (although at least some instances are undoubtedly the result of scavenging)^10,11^.

**Figure 2:**
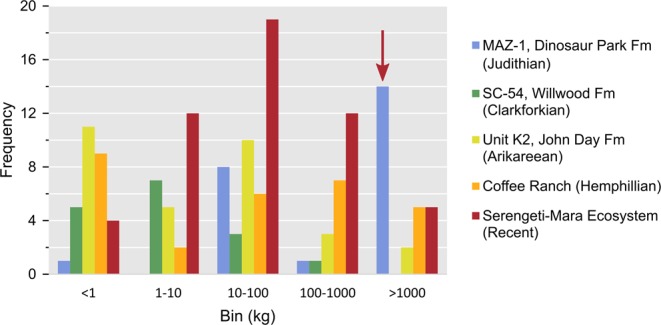
Body size frequency of various herbivore communities, past and present. Note the higher number of megaherbivores (>1,000 kg) in the Dinosaur Park Formation (indicated by red arrow) relative to the mammal assemblages. Data from^26,195–200^. Abbreviation: Fm, Formation.

The community ecology, and particularly the coexistence of its constituent species, has proved especially perplexing as it relates to the megaherbivorous dinosaur assemblage of the Late Cretaceous island continent of Laramidia. This diminutive landmass (4 million–7.7 million km^2^ ^12,13^) resulted from the flooding of the North American Western Interior between approximately 100 and 66 million years ago. Megaherbivorous ankylosaurs, ceratopsids, and hadrosaurids were particularly abundant here, and account for the majority of the fossil assemblage, in terms of both diversity and biomass. The megaherbivore diversity of various well-sampled terrestrial mammal assemblages pales by comparison, even given larger habitable areas^14^ (Fig. 2). The problem of megaherbivore coexistence on Laramidia is further compounded by the large nutritional requirements of these animals^14–16^, and their high population densities^17–19^, which would have placed increased pressure on the resource base.

Predatory dinosaurs were common on Laramidia^20^, but only the large (2,000–8,000 kg) tyrannosaurids were capable of felling the megaherbivores. However, whether they did so with such frequency as to shape the structure of the megaherbivore assemblage is unclear. If the megaherbivorous dinosaurs were instead resource-limited, as contended here, then the following argument might be proffered:

P1. If dietary resources on Laramidia were limiting to megaherbivores, then species overlap in ecomorphospace should have been minimized, which would have reduced resource competition and facilitated dietary niche partitioning among sympatric species.

P2. Where like ecomorphotypes did coexist, their sympatry should have been short-lived or implicated only rare taxa, due to competitive constraints.

P3. Overlap in ecomorphospace was limited.

P4. Similar ecomorphotypes were incapable of prolonged coexistence.

C: Therefore, dietary resources were limiting and competition regulated megaherbivore diversity on Laramidia.

In this syllogism, P1 and P2 follow from Gause’s competitive exclusion principle^21^. P1 assumes that ecomorphotype reflects niche occupation, which is generally well-supported^22^. P1 and P3 are insufficient to run the argument on their own because it is possible that negligible overlap in ecomorphospace can occur stochastically via migration, despite any underlying organizing principles^23^; the multidimensionality of niche hyperspace is such that the probability that any two species will occupy the same space is low due to chance alone^24^. Therefore, to demonstrate the effect of competition for limited resources, it is also necessary to show that like ecomorphotypes cannot coexist indefinitely (P2), owing to the preoccupation of niche space. The remainder of this paper will focus on demonstrating P3 and P4, which are simply the manifestations of P1 and P2, respectively.

## Materials and Methods

### Model system

**Figure 3:**
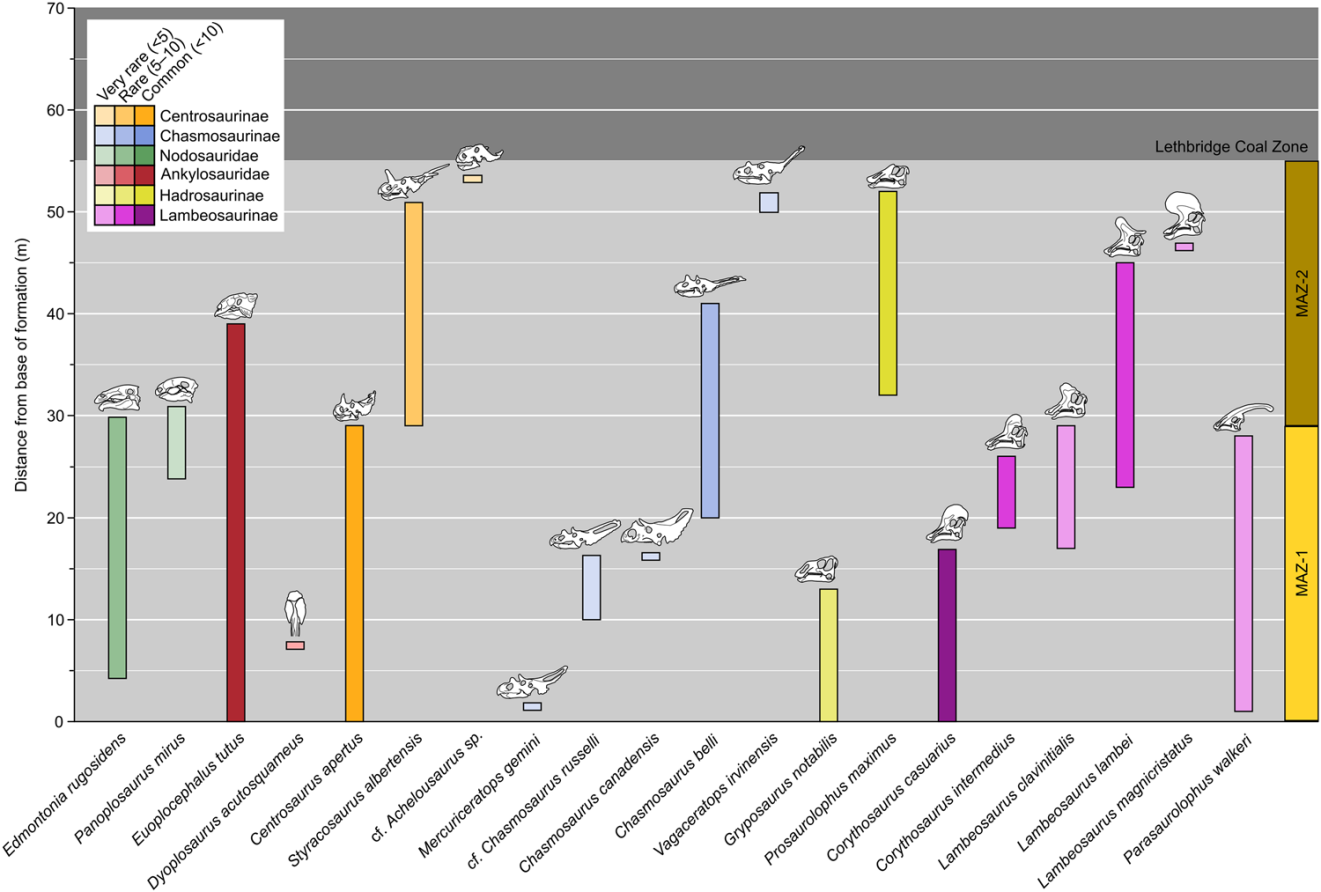
Biostratigraphy of megaherbivores from the Dinosaur Park Formation, in the area of Dinosaur Provincial Park, Alberta. Note that stratigraphic overlap between members of the same family (in the case of ankylosaurs) or subfamily (in the case of ceratopsids and hadrosaurids) is limited, only involving examples of rare or short-lived species. Abbreviations: MAZ, Megaherbivore Assemblage Zone. Modified from Mallon *et al*.^29^.

Laramidia stretched from what is now the North Slope of Alaska to Mexico. Dinosaur assemblages from intermontane, alluvial and coastal plain deposits have been found throughout this ancient landmass, but among the best sampled and consequently most diverse is that of the Dinosaur Park Formation (DPF, middle to upper Campanian) of Alberta, Canada. The DPF is an alluvial-coastal plain deposit that records the third order transgressive event of the Western Interior Seaway (Bearpaw phase). Recent estimates, based on ^40^Ar/^39^Ar dating, place the lower boundary of the DPF at ~77 Ma and the upper at ~75.5 Ma^25^. At present, over 30 herbivorous and omnivorous dinosaur species are recognized from the DPF^26^. Ongoing biostratigraphic work has shown that these taxa were not all contemporaneous, but that different species are restricted to different horizons within the formation^27,28^ (Fig. 3). Mallon *et al*.^29^ used ordination and clustering methods to divvy the megaherbivore assemblage of the DPF into two zones, the older Megaherbivore Assemblage Zone 1 (MAZ-1) and the younger Megaherbivore Assemblage Zone 2 (MAZ-2). Despite minor discrepancies, these closely matched the zones previously identified by Ryan and Evans^27^ based on a qualitative assessment of species distribution. Establishment of the MAZs limits the confounding effects of time-averaging to a scale of approximately 600 Kyr^29^, while creating an objective and meaningful framework in which to consider ecological interactions within the DPF megaherbivore assemblage. The representative fauna, high biodiversity, and exceptional stratigraphic control thus combine to make the DPF an ideal model system for studying species interactions during the Late Cretaceous of Laramidia. These conditions are not currently met elsewhere in the fossil record of the North American Western Interior or, indeed, elsewhere in the dinosaur fossil record.

### Analysis

The megaherbivore assemblage of the DPF has been the subject of intense interest lately, particularly as it relates to the matter of niche partitioning. Recent studies of this assemblage (primarily stemming from the PhD research of Mallon^30^), have examined variability in feeding height^31^, skull and beak morphology^32,33^, tooth wear^34^, and jaw mechanics^35^. In order to discern more completely the matter of megaherbivore niche partitioning, data from these studies were combined into a single meta-analysis. Body mass is also of great ecological importance^5,36^, and was estimated for each specimen, where possible. This was done using the R package MASSTIMATE^37^, which estimates body mass using the limb bone scaling formula of Campione and Evans^38^. Twenty-one variables were considered in total (Table 1). To minimize the amount of missing information while maximizing the size of the dataset, only those specimens with ≥50% missing data were included, for a total of 75 specimens representing 14 genera. The complete 21 × 75 matrix is available in Supplementary Data S1.

**Table 1:** Variables used in this study.

	Variable
1	Maximum feeding height (MaxFeedH)^31^
2	Beak shape PC 1 (BeakPC1)^33^
3	Beak shape PC 2 (BeakPC2)^33^
4	Minimum relative bite force (MinRBF)^35^
5	Maximum relative bite force (MaxRBF)^35^
6	Distance from jaw joint to anterior beak tip (SkullL1)^32^
7	Distance from jaw joint to posterior edge of beak (SkullL2)^32^
8	Distance from jaw joint to anterior end of tooth row (SkullL3)^32^
9	Distance from jaw joint to posterior end of tooth row (SkullL4)^32^
10	Maximum beak width (BeakW)^32^
11	Facial height, measured from base of tooth row to dorsal surface of orbit (FaceH)^32^
12	Occiput height, measured from ventral edge of foramen magnum to dorsal edge of occiput (OccH)^32^
13	Paroccipital process breadth, measured as the sum of the lengths of the left and right paroccipital processes (ParaL)^32^
14	Distance between quadrates (QuadDist)^32^
15	Depression of snout below occlusal plane (SnoutPos)^32^
16	Dentary height, measured at midpoint of tooth row (DentH)^32^
17	Distance from jaw joint to coronoid process apex (JCP)^32^
18	Microwear average scratch count (MWAvgS)^34^
19	Microwear average pit count (MWAvgP)^34^
20	Microwear average feature width (MWAvgWidth)^34^
21	Body mass (Mass)

Abbreviations correspond to entries in Supplementary Data S1.

To assess the extent to which different taxonomic groups occupied ecomorphospace, non-metric multidimensional scaling (NMDS) was used to ordinate the data with the metaMDS() function in the vegan package^39^ for R v. 0.99.902^40^. NMDS was chosen because it is robust to missing data and can handle mixed variable datasets on account of its use of ranked distances over the original distance values. The variables were both left untransformed and subjected to z-score transformation to equalize their weights^41,42^. Arguably, the former approach better captures the ecological relationships of the taxa considered here because it does not suppress those variables that have a dominant effect on niche differentiation (e.g., body mass)^5,36^. A Euclidean distance metric was used in the calculation of the initial dissimilarity matrix, and dimensionality (k) was allowed to vary between k = 2 and k = 4 to assess its influence on morphospace reconstruction. Because NMDS is easily trapped on local optima, the analysis was run iteratively using a minimum of 10,000 random starts until two convergent solutions were reached (no convergent solutions were reached at k > 4 for the untransformed data). Unlike the more conventional principal component analysis, NMDS does not yield eigenvectors; rather, its axes are arbitrary, and variable loadings on those axes cannot be deduced^43^. The relationships of variables to axes were instead estimated using Kendall’s tau rank correlation^41,44^.

The distribution of the data was considered at different taxonomic (family/subfamily + suborder/family + genus) and temporal (time-averaged/MAZ-1/MAZ-2) scales to seek compromise between resolution and sample size. Numerous studies^31–35^ were unable to demonstrate significant ecomorphological differences at the species level, so those differences were not investigated here. Comparisons were also made of taxa between MAZ-1 and -2 to assess changes in ecomorphospace occupation through time. Significant (α = 0.05) taxonomic and temporal differences in NMDS scores were sought using one-way permutational multivariate analysis of variance (PERMANOVA), and post-hoc pairwise comparisons were made with and without partial Bonferroni correction. Statistical comparisons and correlations were conducted in PAST v. 3.15^45^.

## Results

### Time-averaged ecomorphospace

**Figure 4:**
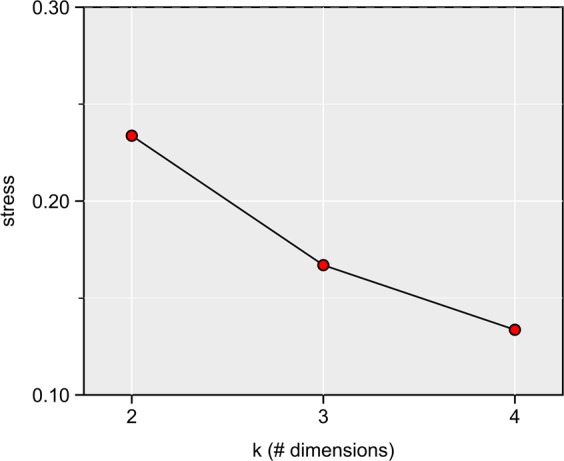
NMDS scree plot showing stress for different values of k.

**Figure 5:**
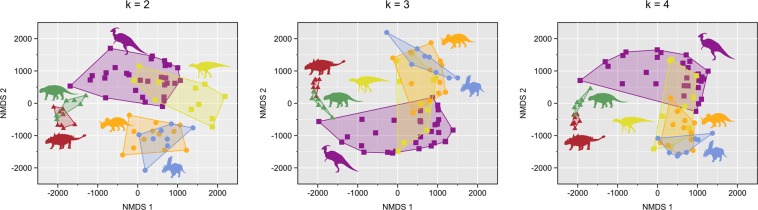
Time-averaged ecomorphospace for different values of k. Colour scheme: red, Ankylosauridae; green, Nodosauridae; orange, Centrosaurinae; blue, Chasmosaurinae; yellow, Hadrosaurinae; purple, Lambeosaurinae. Silhouettes not to scale.

**Figure 6:**
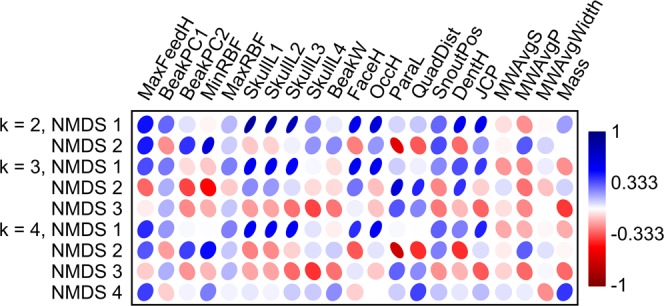
Kendall’s tau correlation map showing relationships between NMDS scores and the 21 ecomorphological variables used in this study. Abbreviations after Table 1.

The results obtained here are broadly consistent with those reported elsewhere using subsets of the current dataset^31–35^. Likewise, the results stemming from the untransformed data largely agree with those from the transformed data, and so only the former are reported here. Full statistical details are provided in Supplementary Data S1 (untransformed data) and S2 (transformed data). NMDS stress values vary from poor (0.23) at k = 2 to good (0.13) at k = 4 (Fig. 4), although the ordination results are all generally comparable. At all values of k, there is statistical support for taxonomic separation in the time-averaged ecomorphospace (*p* < 0.0001). Pairwise comparisons show that Ankylosauria, Ceratopsidae, and Hadrosauridae are all significantly distinct from one another (*p* < 0.0001 for all k), and there is limited or no overlap between them in ecomorphospace (Fig. 5). Ankylosaurs consistently plot well apart from ceratopsids along the first NMDS axis (most strongly and positively correlated with metrics of facial length, skull height, and coronoid process height; Fig. 6) with hadrosaurids (particularly lambeosaurines) spanning the distance between them. Ceratopsids are best separated from hadrosaurids along the second axis (most strongly correlated with metrics of posterior skull breadth, minimum relative bite force, and beak shape; Fig. 6), with some overlap occurring at higher values of k. Ankylosaurs plot between them. At k = 3 and k = 4, there is some overlap of Ceratopsidae and Hadrosauridae (particularly Hadrosaurinae) along the second axis (Fig. 5).

There is generally good taxonomic separation even within these higher level taxa (Table 2). For all values of k, ankylosaurids and nodosaurids are well separated along the second NMDS axis, with minimal overlap (Fig. 5), although statistical significance is manifest only at k = 2 and k = 3 (*p* < 0.05, uncorrected), likely reflective of small sample size. Hadrosaurines and lambeosaurines also occupy distinct (*p* < 0.001 for all k), yet overlapping, areas of morphospace; they are best separated along the first (for k = 2) or second (for k = 3 and k = 4) axes. The separation of centrosaurines and chasmosaurines is weak; the two groups are statistically distinguishable (*p* < 0.05, uncorrected) only at k = 4, although the *p*-value at k = 3 approaches significance (Table 2), and all *p*-values are likely affected by small sample size. Chasmosaurines plot more distally on axis 2 than centrosaurines, particularly at higher values of k (Fig. 5). Taxonomic separation is reduced along higher NMDS axes (i.e., 3 and 4; Supplementary Data S1), and axis score correlation with the original ecomorphological variables is correspondingly depressed (Fig. 6). Additionally, there is statistically poor separation of genera within the ankylosaur families, and within the ceratopsid and hadrosaurid subfamilies (Supplementary Data S1).

**Table 2:** Family and subfamily pairwise comparisons for the time-averaged analysis.

	Ankylosauridae	Nodosauridae	Centrosaurinae	Chasmosaurinae	Hadrosaurinae	Lambeosaurinae
k = 2, F = 36.61, *p* < 0.0001						
Ankylosauridae		**0.0318**	**<0.0001**	**3.00 × 10** ^**−4**^	**<0.0001**	**<0.0001**
Nodosauridae	0.0312		**<0.0001**	**<0.0001**	**<0.0001**	**<0.0001**
Centrosaurinae	**<0.0001**	**<0.0001**		0.4973	**<0.0001**	**<0.0001**
Chasmosaurinae	**<0.0001**	**0.0003**	0.4973		**<0.0001**	**<0.0001**
Hadrosaurinae	**<0.0001**	**0.0002**	**<0.0001**	**<0.0001**		**0.0005**
Lambeosaurinae	**<0.0001**	**<0.0001**	**<0.0001**	**<0.0001**	**0.0003**	
k = 3, F = 24.33, *p***<**0.0001						
Ankylosauridae		**0.0253**	**<0.0001**	**0.0003**	**<0.0001**	**<0.0001**
Nodosauridae	0.0253		**<0.0001**	**0.0003**	**0.0003**	**<0.0001**
Centrosaurinae	**<0.0001**	**<0.0001**		0.06339	**<0.0001**	**<0.0001**
Chasmosaurinae	**0.0003**	**0.0003**	0.06339		**<0.0001**	**<0.0001**
Hadrosaurinae	**<0.0001**	**0.0003**	**<0.0001**	**<0.0001**		**<0.0001**
Lambeosaurinae	**<0.0001**	**<0.0001**	**<0.0001**	**<0.0001**	**<0.0001**	
k = 4, F = 9.00, *p***<**0.0001						
Ankylosauridae		0.05129	**<0.0001**	**0.0005**	**<0.0001**	**<0.0001**
Nodosauridae	0.05129		**<0.0001**	**0.0003**	**<0.0001**	**<0.0001**
Centrosaurinae	**<0.0001**	**<0.0001**		**0.0357**	**<0.0001**	**<0.0001**
Chasmosaurinae	**0.0005**	**0.0003**	0.0357		**0.0002**	**<0.0001**
Hadrosaurinae	**<0.0001**	**<0.0001**	**<0.0001**	**0.0002**		**<0.0001**
Lambeosaurinae	**<0.0001**	**<0.0001**	**<0.0001**	**<0.0001**	**<0.0001**	

The overall PERMANOVA statistics are reported at the top. Uncorrected pairwise comparisons are given in the upper right triangle; partial Bonferonni corrected pairwise comparisons are given in the lower left triangle (note: differences in significance between tests reflect corrected α-values, not *p*-values). Significant results are reported in **bold**.

### Temporal comparisons

**Figure 7:**
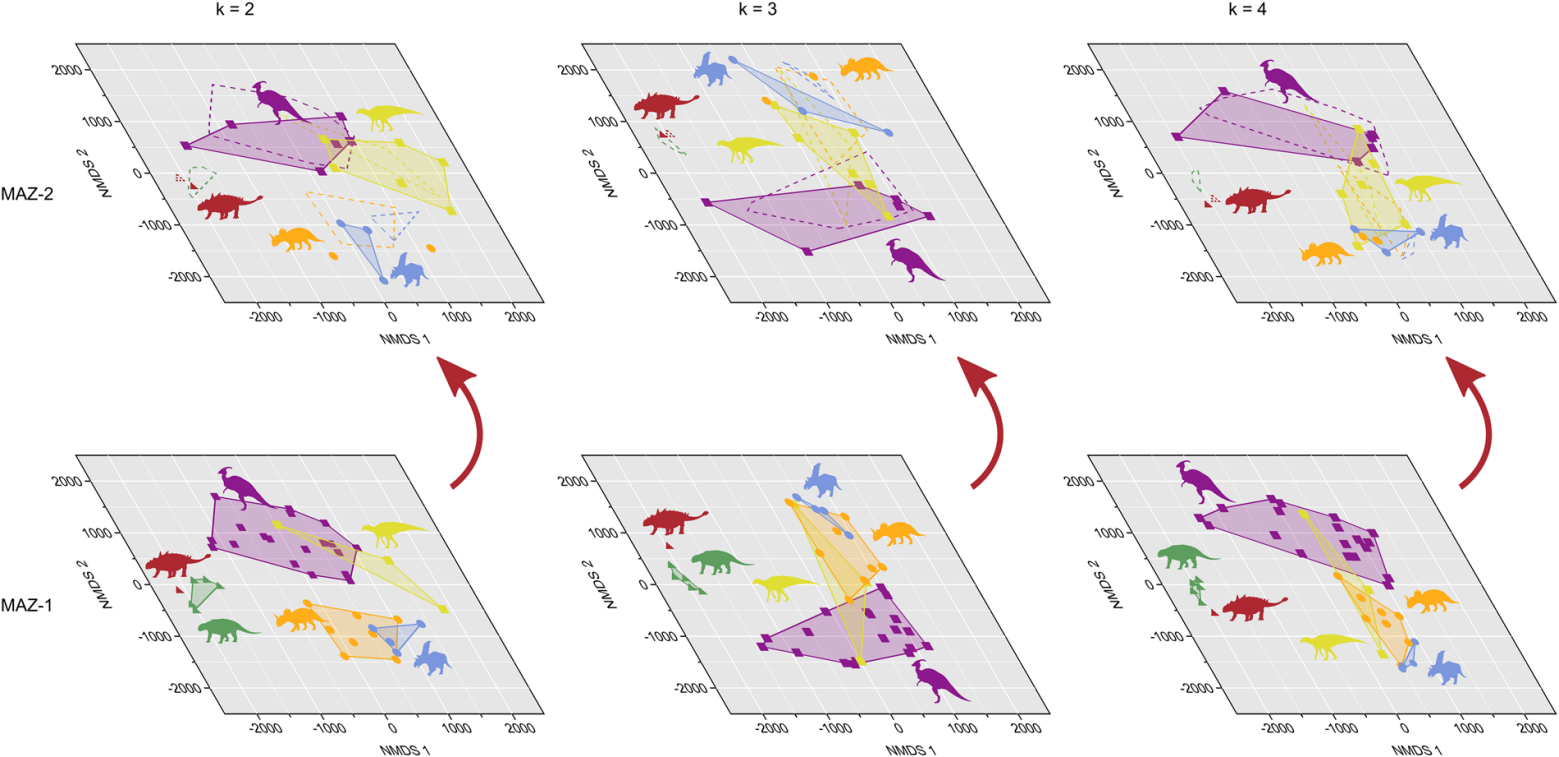
Time-constrained ecomorphospace comparisons between MAZ-1 and -2 for different values of k. Dashed lines in MAZ-2 ecomorphospaces indicate original taxon distribution in MAZ-1. Note that megaherbivore distribution in ecomorphospace varies little through time. Silhouettes not to scale. Colours after Fig. 5.

The patterns observed in the time-averaged sample above are largely stable through time, such that the various ankylosaurs, ceratopsids, and hadrosaurids occupy the same general regions of ecomorphospace between the lower (MAZ-1) and upper (MAZ-2) parts of the DPF, a timespan of approximately 1.5 Myr (Fig. 7). At k = 2, there is a small but significant (N = 7, F = 3.65, *p* < 0.05) shift of Chasmosaurinae along the second axis between MAZ-1 and -2. There are notably fewer individuals sampled within MAZ-2, reflective of taphonomic and sampling biases in the muddy host unit^28^. Nodosaurids are entirely absent from the MAZ-2 sample, and ankylosaurids (N = 1) and centrosaurines (N = 2) are likewise rare.

Further insight can be gained by cross-referencing the biostratigraphic distribution of megaherbivores in the DPF with the ecomorphological patterns observed in MAZ-1 and -2. At any horizon within the DPF, there is typically only a single contemporaneous species of Ankylosauridae, Nodosauridae, Centrosaurinae, Chasmosaurinae, Hadrosaurinae, and Lambeosaurinae (Fig. 3). Thus, the ecomophological patterns and relationships seen in Figs 5 and 7 are likely representative of the megaherbivore standing crop at any given time. Where exceptions to this pattern occur, they typically involve very rare (e.g., *Panoplosaurus mirus*, *Dyoplosaurus acutosquameus*, *Chasmosaurus canadensis*, *Lambeosaurus clavinitialis*, *Parasaurolophus walkeri*) or short-lived (e.g., *Corythosaurus intermedius*) species (Fig. 3).

## Discussion

### Palaeoecology of the DPF megaherbivore assemblage

If resources were limiting during the Late Cretaceous of western North America, there should be minimal evidence for significant ecomorphological overlap among sympatric megaherbivores. Or, where overlap occurs, it should be short-lived on account of competitive exclusion. Both of these premises (P1 and P2 in the introductory syllogism) are borne out in this study (P3 and P4), suggesting both that competition had a role in shaping the megaherbivore community structure of the Late Cretaceous, and that the high megaherbivore standing diversity (richness) reported here was enabled through niche partitioning. This is not a trivial discovery; it has been hypothesized that the high megaherbivore diversity on Laramidia resulted from (non-limiting) resource abundance, due to elevated primary productivity^46^, to the exceptionally low metabolic requirements of dinosaurs^12,16^, or both^46,47^. Predation and disease are also capable of reducing herbivore abundance and alleviating pressure on the resource base^48,49^, but these mechanisms have not been implicated in the structuring of dinosaur communities. Regardless, none of these hypotheses predict the structural patterns noted here. Although dietary niche partitioning has been posited in other ancient, non-mammalian communities^42,50,51^, this study is among the first to demonstrate its operation within a single assemblage using a meta-analytic approach. Further evidence for resource limitation during the Late Cretaceous stems from a consideration of ecological energetics^14,52^.

Ankylosaurs and ceratopsids appear to have been most ecologically distinct, given how far they plot from one another in the NMDS analyses. In a sense, this might be surprising: ankylosaurs and ceratopsids are both large, obligate quadrupeds that fed low to the ground^31^, so one might expect that they would share similar ecologies, particularly when compared to the facultatively quadrupedal hadrosaurids. Nevertheless, there also exists a bevy of ecomorphological differences between the first two taxa. Ankylosaurs had smaller, relatively wider skulls with broader muzzles that would have allowed them to feed less selectively^32,33^. Ankylosaur dentaries were relatively deeper than those of ceratopsids, yet were capable of producing far lower bite forces^35^. Their tooth rows were shorter, and the teeth were simple and loosely arranged compared to the elongate and complex dental batteries of the ceratopsids, whose dentition was strictly limited to shearing, as reflected by the microwear^34^. These functional differences allowed ankylosaurs and ceratopsids to partition the low browsing guild.

The fact that hadrosaurids share certain features in common with ankylosaurs (e.g., wide muzzles^33^, higher dental microwear pit counts^34^) and others in common with ceratopsids (e.g., large and elongate skulls^32^, elevated bite forces^35^, tooth batteries^34^), and the fact that they exhibit a particularly large area of ecomorphospace, suggests that hadrosaurids had comparatively catholic herbivorous diets. Indeed, it is reasonable to suspect that they may have occasionally shared resources with both ankylosaurs and ceratopsids. Nevertheless, hadrosaurids were quite distinct in that they could feed at heights of up to 5 m above ground^31^, and their teeth were capable of crushing, grinding, and shearing^34^, enabling these animals access to plants not available to the other megaherbivores. In light of their particularly large sizes, generalist diets (see ‘Megaherbivorous dinosaur diets’ below), and propensity to form herds^18,53,54^, hadrosaurids may have had the greatest impact on structuring plant communities and the smaller fauna that inhabited them. In this sense, hadrosaurids might have served as ecosystem engineers^55^, much like modern elephants^56,57^.

Even within these higher level taxa, there is evidence for niche partitioning, particularly between members of different families and subfamilies. Ankylosaurids and nodosaurids were both restricted to low browsing, yet the former differed from the latter in having wider, squarer beaks^33^, weaker jaws^35^, and smaller, more cusp-like teeth^34,58^. Hadrosaurines and lambeosaurines were able to exploit a wider range of vegetation strata^31^, and could therefore divvy food resources in that way. Hadrosaurines are further distinguishable from lambeosaurines in having larger skulls with less ventrally deflected beaks^32^, and more numerous, but finer microwear scratches^34^. The evidence for niche partitioning between centrosaurines and chasmosaurines is slim, but statistically significant (Table 2), in spite of the fact that previous studies failed to find significant ecomorphological differences between the two groups. In this case, it appears that near-significant results from those previous studies multiplicatively combined to yield significance here. Thus, although Mallon and Anderson^32^ could not demonstrate that centrosaurines have deeper skulls than chasmosaurines (*p* = 0.077), and Mallon and Anderson^34^ could not quite show that centrosaurines have statistically higher microwear scratch counts than chasmosaurines (*p* = 0.051), the synthesis of these data in a larger meta-analysis more convincingly establishes the distinct ecomorphologies of the two groups. This point echoes that made by Fraser and Theodor^59^ concerning ungulate dietary proxies.

There is no statistical evidence for niche partitioning below the family level among ankylosaurs or below the subfamily level among ceratopsids and hadrosaurids. This insignificance is not a simple upshot of low sample size because genera within these clades overlap considerably in morphospace (but not temporally).

### Megaherbivorous dinosaur diets

In light of the evidence for dietary niche partitioning presented here, the question naturally arises as to what the megaherbivores of the DPF ate. Redundancy in the form-function complex^60^, lack of modern analogues, and incomplete knowledge of Late Cretaceous terrestrial environments in North America greatly hamper efforts to reconstruct the dietary habits of these dinosaurs. This has not prevented anyone from trying (Table 3). What follows is an attempt to say something constructive about the diets of the DPF megaherbivores, using a total evidence approach. To the extent that the ecomorphotypes considered here are not descriptive of clade members that occur outside the DPF (e.g., narrow-snouted, non-ankylosaurine ankylosaurids^58^), or that other fossil assemblages contain potential foodstuffs not present in the study area (e.g., palms, cycadophytes), these comments do not apply. The inferences are necessarily broad, owing to the incomplete nature of the fossil record. Dietary subtleties between closely related species, or that vary with geography or seasonality, are wholly ignored.

**Table 3:** Non-exhaustive survey of inferred foodstuffs for megaherbivorous dinosaurs present in the Dinosaur Park Formation.

Taxon	Food
all	gymnosperms, ferns and other “pteridophytes“^162^
Ankylosauria	insects (locusts)^163^
	succulent ground vegetation^164^
	soft or juicy vegetable matter^165^
	less abrasive, more nutritious plants^15^
	plants, insects, carrion^166^
	ferns, lilies, arum plants, cattail tubers^167^
	low-fibre food^91^
	ferns and horsetails^168^
	soft, aquatic vegetation^169^
	bennettitalian inflorescences, angiosperm fructifications^170^
	leaves^66^
	soft plant food^171^
	ferns^172^
	fibrous or vascular tissue (leaves), angiosperm fruits or endocarps, small seeds, and fern sporangia^71^
	tough foodstuffs^173^
	cycad seeds^87^
	horsetails^82^
	ferns and cycadophytes^174^
	ferns and fern allies^103^
Ankylosauridae	herbs^175^
	low-growing vegetation^74^
Nodosauridae	low-growing, woody vegetation^175^
	leaves^74^
Ceratopsidae	leaves and shoots of low trees and shrubs^176^
	ferns, cycads, equisetae, and other luxuriantly crowned vegetation^177^
	succulent roots^178^
	cycads and palms^80,179^
	fibrous plants^15^
	reed and cattail tubers^167^
	toughest, low-growing shrubs^91^
	low-growing, woody vegetation (fronds or branches)^175^
	cycadeoid bennettite fronds and strobili, fruits^168^
	bennettitalian and angiosperm fructifications^170^
	cycadeoid fronds^66^
	fibrous plant material^180^
	angiosperm trees^83–85^
	cycadophytes^172^
	ferns^181^
	ferns and cycadophytes^174^
	cycad stems^182^
	palm seeds and fruits^183^
Hadrosauridae	soft, aquatic vegetation^184^
	*Cunninghamites elegans* (conifer) needles, conifer and deciduous branches, small seeds or fruits^101^
	primarily equisetalians, occasional herbaceous vegetation, roots of water lilies and other aquatic plants (analogy to moose)^185^
	resistant, fibrous, woody plants^46^
	mollusks, small crustaceans, aquatic plants^186^
	fibrous, siliceous, or woody plants^15^
	ferns, fresh leaves and shoots^167^
	high-fibre food^91^
	unspecialized browse^175^
	drifted plant materials or peat^168^
	low-growing herbs, leaves and twigs of angiosperm trees, lush seasonal water plants^169^
	ginkgophyte, conifer, nilssonalian, and angiosperm fructifications^170^
Hadrosauridae	horsetails, ground pine, ferns, low tree ferns, seedling evergreens (pines, cypress, etc.), cycads and other tough-frond types, low-growing palms, magnolialike shrubs^66^
	leaves and small stems of angiosperm herbs^83^
	young gymnosperms and angiosperms (wood, seeds, and seed pods), charcoal^98^
	conifer wood^99^
	low-quality, high-fibre vegetation (foliage and fructifications)^187^
	fungally decayed conifer wood^100^
	ginkgos and conifers^174^
	palm seeds and fruits^183^
	conifers^172^
	mostly leaves^102^
	*Equisetum* ^188^
	crustaceans^104^
Hadrosaurinae	open-habitat browse^41^
Lambeosaurinae	closed-habitat browse^41^

#### Ankylosauria

Because of their small and supposedly weak teeth, ankylosaurs have traditionally been attributed a diet of soft, possibly aquatic plants (Table 3). However, this seems unlikely for two reasons. First, distribution data indicate that ankylosaurs may have preferred inland settings^29,61,62^ and probably did not habitually dwell in the wet, swampy coastal plain settings where aquatic plants were most common (but see Butler and Barrett^63^). Second, ankylosaurs exhibit a suite of features suggestive of a more resistant plant diet, including a transversely wide skull, deep mandible with dorsally bowed tooth row^32^, and ossified secondary palate^64,65^, all of which would have dissipated stress associated with resilient foodstuffs. The phylliform teeth of ankylosaurs, although similar to those of iguanines in shape, are also heavily worn, indicating that ankylosaurs were more effective at comminuting plant material than their lepidosaurian counterparts^34^. Finally, the laterally expanded gut of the ankylosaurs would have increased both retention time and the space available for cellulolytic microflora, further aiding in the breakdown of resistant plants^66^.

In fact, given differences in the cranial anatomy of ankylosaurids and nodosaurids, ankylosaur diets were probably more varied than traditionally assumed. Ankylosaurids (represented in the DPF by *Euoplocephalus tutus* and the rare *Anodontosaurus lambei* and *Dyoplosaurus acutosquameus*) are interpreted here as consumers of low-growing ferns (Polypodiales), as evidenced by several features. First, all ankylosaurs were restricted to feeding at heights < 1 m above the ground^31^, and must therefore have browsed within the herb layer. Second, the wide, square beaks of ankylosaurids^33^ are suggestive of high intake rates typically associated with consumers of low-quality (high-fibre) vegetation. The data of Hummel^67^ indicate that, compared to most other vascular plants, ferns are less nutritious, being lower in metabolizable energy and higher in fibre. They therefore seem likely as ankylosaurid fodder. Ferns and other ‘pteridophytes’ from the DPF comprise ~39% of the total palynofloral diversity^68^, and include examples of Osmundaceae (cinnamon ferns), Schizaeaceae (climbing ferns), Gleicheniaceae, and Cyatheaceae, among others^69^. The interpretation of ankylosaurids as fern consumers agrees with a cololite found inside the gut of a small (~300 kg^70^), Early Cretaceous ankylosaurid from Australia^71^. In addition to an abundance of vascular tissue (possibly leaves), the fossil contains angiosperm fruits, small seeds, and probable fern sporangia. It is likely that, on account of their much larger size (~2,300 kg^72^), the DPF ankylosaurids could tolerate low-quality fern material in much greater proportion.

One way that nodosaurids (represented in the DPF by *Panoplosaurus mirus* and *Edmontonia rugosidens*) differ from ankylosaurids (particularly Late Cretaceous ankylosaurines) is in having narrower, more rounded beaks^33,58,73–76^. Nodosaurids therefore appear to have been more selective than these ankylosaurids, and probably consumed more nutritious vegetation, such as shrubby dicotyledonous (dicot) browse. This interpretation would also account for other aspects of nodosaurid morphology. For example, the taller coronoid process suggests the ability to generate higher bite forces possibly associated with a diet of woody browse^32,35,58^, and the distally dilated process of the vomers may have dissipated stresses associated with those higher bite forces^77,78^. The mesiodistally expanded, bladed teeth of nodosaurids also suggest an incipient ability to cope with the crack-stopping mechanism of woody plant material^34,79^. To be sure, nodosaurids do not share the same highly-modified morphology of ceratopsids for rending woody browse (see Ceratopsidae below), so it is unlikely that nodosaurids fed exclusively on this type of vegetation. Instead, both ankylosaurids and nodosaurids likely consumed mostly herbaceous ferns, with the latter supplementing their diet with dicot browse. The relatively high number of dental microwear pits in both these taxa indicates that they may also have eaten fruits or seeds on occasion^34^.

#### Ceratopsidae

Several lines of evidence point to the fact that ceratopsids subsisted on particularly tough vegetation. The skull was massive—more so than in any other animal from the DPF—indicating that these animals could generate absolutely high bite forces^32^. The lower jaw was also constructed in such a way as to produce relatively high bite forces^35^. The triangular beak was narrow, and could be used to selectively crop coarse plant matter^33^, a feeding strategy shared with black rhinoceroses (*Diceros bicornis*). Yet, probably more than any other feature, the shearing dentition of ceratopsids has played a key role in the inference to their diets. It is commonly and correctly understood that their dentition was particularly suitable to fracturing the toughest plant tissues^34,80,81^. Among the plant foods most regularly attributed to ceratopsid diets are cycadophytes and palms (Arecaceae; Table 3); the fronds of the former are very fibrous and not particularly nutritious^82^. However, probably neither of these was consumed by ceratopsids from the DPF for the simple reason that compelling evidence for the existence of these plants in the formation is lacking^69,83–86^. Furthermore, living cycads are particularly toxic^87,88^, and it is likely that their fossil forbearers were as well. Therefore, even if cycads were available, they were probably not frequently eaten.

In light of these considerations, Dodson^83–85^ suggested that ceratopsids most regularly consumed woody angiosperms. This seems like a more reasonable interpretation for various reasons. First, angiosperms were widespread by the Late Cretaceous, and were particularly abundant in the floodplain environments^89^ that ceratopsids are known to have frequented^61,62,90^. Second, it is likely that angiosperms from the DPF were herbaceous or shrubby in habit because angiosperm wood corresponding to trees is unknown from the formation^69^. These flowering plants were therefore easily within reach of the ceratopsid cropping mechanism^31^. Third, the woody branches and twigs of angiosperm shrubs would have required a bladed dentition for fracture^79^, and the ceratopsid dental battery appears optimally suited to the task^34,81^. Common angiosperms in the DPF include relatives of maples (Aceraceae), beeches (Fagaceae), elms (Ulmaceae), lilies (Liliaceae), sedges and reeds (Cyperaceae), among others^69^. Conifer (Pinales) and ginkgo (Ginkgoaceae) saplings may have been consumed as well, though probably less frequently, given their generally slower replacement rates^88^. Although some^66,91^ have posited coevolutionary scenarios between early angiosperms and ornithischian dinosaurs (including ceratopsids), there is no solid evidence for this^92,93^.

Possible evidence for dietary niche partitioning between coexisting centrosaurines and chasmosaurines comes by way of their different skull proportions and dental microwear signatures; centrosaurines appear to have slightly shorter, deeper crania than chasmosaurines^32^, and higher microwear scratch counts^34^. These data are consistent with the interpretation that centrosaurines ate tougher, more fibrous vegetation than chasmosaurines, requiring more oral processing^94^. The high-angled slicing surfaces of centrosaurine predentaries^13,95^ further support this interpretation.

#### Hadrosauridae

Previous attempts at inferring hadrosaurid diets have varied widely (Table 3). Accordingly, hadrosaurids are interpreted as having been the least selective of the megaherbivores from the DPF; they likely subsisted on a broad range of plant tissues. Evidence for this comes by way of their large body sizes^96^ (which typically equates to a broad dietary range^5^), correspondingly large feeding envelopes^31^, intermediate beak morphologies^32,33^, efficient jaw mechanics^35^, and complex tooth batteries capable of crushing, grinding, and shearing functions^34^. Hadrosaurids also appear to have been most cosmopolitan in their distribution along the alluvial-coastal plain^62,90^, and were therefore tolerant of a wide range of habitats.

Although hadrosaurids could have effectively eaten any plants within reach, it makes sense that they would have preferred more digestible plants in order to maximize their energy intake and meet their large nutritional requirements. Hummel *et al*.^82^ provide data on Mesozoic plant digestibility, derived from living relatives of fossil taxa. Accounting for such limiting factors as temporal and geographic availability, and growth height, hadrosaurids most likely favoured horsetails (*Equisetum* spp.), forbs, ginkgo and conifer (pines, cypresses, and cheirolepids) saplings, and dicot browse, which were the most nutritious plants available. Hummel *et al*.^82^ suggested that horsetails were unlikely fodder for dinosaurs that chewed their food (e.g., ceratopsids and hadrosaurids) because the high silica content would have produced excessive tooth wear. However, the rapid tooth replacement rates of these animals, on the order of every 50–80 days^97^, probably would have offset this problem.

Reported examples of hadrosaurid gut contents^9,97,98^ include abundant conifer material, although the origin of this material (whether autochthonous or not) remains questionable. However, coprolites attributable to hadrosaurids^99,100^ also contain abundant conifer material (including fungally decayed wood), and it is likely that these plants formed a staple of hadrosaurid diets. Angiosperm seeds and fruits also have been reported in hadrosaurid gut contents^98,101^, as well as unidentified leaf material^102^. Bearing in mind the difficulties associated with interpreting these fossils^103^, these contents lend credibility to the interpretation of hadrosaurids as generalist browsers, perhaps even occasionally including animal protein in their diets^104^.

Coexisting hadrosaurines and lambeosaurines from the DPF differ markedly in the development of their cranial ornamentation^27,105^, but the morphological differences that distinguished their feeding ecologies are somewhat more subtle. To this end, hadrosaurines had relatively larger and more protracted skulls than lambeosaurines, with less ventrally deflected beaks^32,106^. These features may suggest that lambeosaurines spent more time feeding on herbaceous vegetation near to the ground, while hadrosaurines habitually extended their long jaws into the boughs of shrubs and trees. Given their broad feeding envelopes^31^, these subfamilies were certainly capable of avoiding niche overlap in this way. Dental microwear evidence further supports this reasoning; scratches on the teeth of the hadrosaurine *Prosaurolophus maximus* are fewer and finer than those of the contemporaneous lambeosaurine *Lambeosaurus lambei*^34^, which correlates with higher browsing in living ungulates^107^.

### Spatiotemporal patterns of the Laramidian megaherbivore assemblage

To what extent were the patterns and processes that operated within the DPF megaherbivore assemblage representative of other assemblages in Laramidia? This question may be considered from both spatial and temporal perspectives.

A recent review of the geochronology of the Western Interior of North America by Fowler^25^ suggests that the Judithian (middle to upper Campanian) DPF is penecontemporaneous with the fossiliferous upper Two Medicine and upper Judith River formations in Montana, the lower to middle Kaiparowits Formation in Utah, and the middle Aguja Formation in Texas. Of these, the local biostratigraphy has been presented for the first three formations^25,108–110^. Although the megaherbivore assemblages from each of these formations are relatively poorly sampled and not necessarily time-equivalent, they are nonetheless close enough to warrant comparison. Examination of the respective assemblages reveals the same predictable suite of sympatric ankylosaurids and nodosaurids, centrosaurines and chasmosaurines, and hadrosaurines and lambeosaurines reported in the DPF (albeit, with different representative species, where determinable) (Table 4). Typically, there is, at most, only one common representative of these taxa; where there are two or more representative species, all but one at most are rare. Thus, the megaherbivore community structure noted in the DPF does not appear to be spatially restricted within Laramidia.

**Table 4:** Megaherbivore community structure through the Late Cretaceous.

NALVA	Time	Province/State	Stratigraphic unit	Ankylosauridae	Nodosauridae	Centrosaurinae	Chasmosaurinae	Hadrosaurinae	Lambeosaurinae	Sauropoda
Aquilian	late Santonian	Alberta	Milk River Fm	indet.	indet.	?	?	indet.	?	—
Judithian	middle Campanian	Alberta	upper Foremost Fm (below Taber coal zone)	indet.	indet.	Xenoceratops foremostensis*	?	indet.	?	—
			Oldman Fm	indet.	indet.	*Coronosaurus brinkmani*	indet.	Brachylophosaurus canadensis** *Hypacrosaurus stebingeri*	—	—
			MAZ-1a, Dinosaur Park Fm	Euoplocephalus tutus* Dyoplosaurus acutosquameus** *Scolosaurus cutleri*?**	Edmontonia rugosidens*	*Centrosaurus apertus*	cf. Chasmosaurus russelli** Chasmosaurus canadensis** Mercuriceratops gemini**	Gryposaurus notabilis*	*Corythosaurus casuarius* Parasaurolophus walkeri**	—
			MAZ-1b, Dinosaur Park Fm	Euoplocephalus tutus*	Edmontonia rugosidens* Panoplosaurus mirus**	*Centrosaurus apertus*	Chasmosaurus belli*	?	Corythosaurus intemedius* Lambeosaurus clavinitialis** Lambeosaurus lambei* Parasaurolophus walkeri**	—
	late Campanian	Alberta	MAZ-2a, Dinosaur Park Fm	Euoplocephalus tutus*	?	Styracosaurus albertensis*	Chasmosaurus belli*	*Prosaurolophus maximus*	Lambeosaurus lambei*	—
			MAZ-2b, Dinosaur Park Fm	?	?	Styracosaurus albertensis* cf. Achelousaurus horneri**	Vagaceratops irvinensis**	*Prosaurolophus maximus*	Lambeosaurus lambei* Lambeosaurus magnicristatus**	
		Utah	lower middle unit, Kaiparowits Fm	Akainacephalus johnsoni**	indet.	Nasutoceratops titusi** n. gen. et sp. (taxon B)**	Utahceratops gettyi* Kosmoceratops richardsoni** Chasmosaurinae n. sp.*	*Gryposaurus* sp.*	*Parasaurolophus* sp.*	—
		Montana	upper Two Medicine Fm	Scolosaurus cutleri*	*Edmontonia rugosidens*	*Einiosaurus procurvicornis* Achelousaurus horneri**	indet.	Prosaurolophus maximus**	*Hypacrosaurus stebingeri*	—
			Coal Ridge Mbr, Judith River Fm	Zuul crurivastator**	indet.	cf. Avaceratops lammersi**	Spiclypeus shipporum** Mercuriceratops gemini**	indet.	indet.	—
Kirtlandian	late Campanian	New Mexico	Willow Wash local fauna, Fruitland Fm	Ziapelta sanjuanensis**	Nodocephalosaurus kirtlandensis**	n. gen. et sp.	*Pentaceratops sternbergi*	Naashoibitosaurus ostromi** Kritosaurus navajovius*	Parasaurolophus tubicen**	*Alamosaurus sanjuanensis*
Edmontonian	late Campanian, early Maastrichtian	Alberta	lower Horseshoe Canyon Fm	Anodontosaurus lambei*	Edmontonia longiceps**	*Pachyrhinosaurus canadensis* **	*Anchiceratops ornatus* Arrhinoceratops brachyops**	*Edmontosaurus regalis* Saurolophus osborni*	*Hypacrosaurus altispinus*	—
Lancian	latest Maastrichtian	Alberta	lower Scollard Fm	Ankylosaurus magniventris**	—	—	Triceratops prorsus* cf. Torosaurus**	?	—	—
		Saskatchewan	Frenchman Fm	?	?	—	Triceratops prorsus* cf. Torosaurus**	Edmontosaurus annectens*	—	—
		Montana	lower (L3) Hell Creek Fm	Ankylosaurus magniventris**	—	—	*Triceratops horridus* Torosaurus latus**	Edmontosaurus annectens*	—	—
			upper (U3) Hell Creek Fm	—	—	—	*Triceratops prorsus*	Edmontosaurus annectens*	—	—

Taxa marked by a single asterisk (*) are rare (5–10 specimens), and those marked by double asterisks (**) are very rare (**<**5 specimens). Question marks (?) denote uncertainty regarding the presence of a particular taxon (e.g., definitive ceratopsid material that cannot be positively attributed to either Centrosaurinae or Chasmosaurinae); dashes (–) denote absence of material that could be justifiably assigned to a particular taxon, given current evidence. Abbreviations: Fm, Formation; Mbr, Member; NALVA, North American Land Vertebrate Age. Data from various sources^26,29,109–111,125–127,130,189–194^.

The time at which the predictable community structure noted here became established remains elusive. The diverse megaherbivore assemblage of the DPF is effectively the earliest such assemblage known from Upper Cretaceous deposits. Slightly earlier deposits (e.g., Milk River, Foremost, and Oldman formations in Alberta, lower Two Medicine and lower Judith River formations in Montana, Wahweap Formation in Utah, lower Aguja Formation in Texas) are comparatively poorly sampled, and although they contain evidence for various ankylosaurs, ceratopsids, and hadrosaurids^111^, the structure of these communities is not well understood.

The megaherbivore community structure exemplified by the DPF evidently evolved in a gradual and piecemeal fashion. Nodosaurids and ankylosaurids date back to the Late Jurassic and Early Cretaceous of North America, respectively, although their points of origin are ambiguous^112^. Their co-occurrence likewise dates to the Early Cretaceous in both Europe and North America^113,114^. The feeding apparatus of both clades is known to have evolved over the ensuing tens of millions of years, as did their presumed ecological roles^58^. Ceratopsids originated in the early to middle Campanian (Late Cretaceous) in North America, which coincides with the first appearance of centrosaurines^115–117^. Chasmosaurines did not appear until the middle Campanian in North America^25,118^, although their ghost lineage presumably extended back to the early Campanian. The first known co-occurrence of centrosaurines and chasmosaurines dates to the middle Campanian^25^. The earliest hadrosaurines are early Campanian in age^119,120^, and the first lambeosaurines date to the late Santonian/early Campanian^121^, both from North America. They may have co-occurred as early as the middle Campanian^111,122^. The earliest known assemblage of ankylosaurs, ceratopsids, and hadrosaurids is from the upper Santonian Foremost Formation of Alberta^123–125^.

Following the deposition of the DPF, the predictable community structure noted here continued essentially intact. The same suite of ankylosaurids, nodosaurids, centrosaurines, chasmosaurines, hadrosaurines, and lambeosaurines was present throughout the Kirtlandian, represented primarily by deposits of the uppermost Fruitland and Kirtland formations in New Mexico^126,127^ (Table 4). Notably, the enormous (ca. 70,000 kg) sauropod *Alamosaurus sanjuanensis* also appeared at this time^128^, possibly an immigrant from South America^129^, but its ecological relationship to the other megaherbivores is unclear.

This community structure continued into the Edmontonian, seen in the lower deposits (uppermost Campanian) of the Horseshoe Canyon Formation of Alberta, where the same typical suite of megaherbivores persisted (Table 4). Megaherbivore diversity appears to wane higher in section, but this is at least partly due to taphonomic biases in the Carbon and Whitemud members^130^. Nonetheless, the loss of both centrosaurines and hadrosaurines around this time (ca. 68 Ma) appears genuine, as these taxa do not postdate this time elsewhere in North America^25^. Interestingly, Brown and Henderson^131^ demonstrated that the chasmosaurine *Regaliceratops peterhewsi* was morphologically convergent on the centrosaurine cranial plan, and it is possible that it filled not only a similar behavioural role to the vacated centrosaurines, but a similar ecological role as well. Although the lower jaws of *R. peterhewsi* are unknown, those of other triceratopsin chasmosaurines that radiated at this time are distinctly longer and lower than those of other chasmosaurines, with taller coronoid processes^132,133^. This new configuration resulted in higher stress production in the lower jaw, but the ecological ramifications that followed are unclear.

The depauperate nature of the megaherbivore fauna of the Lancian (uppermost Maastrichtian) in Laramidia is well documented^134–136^; centrosaurines and lambeosaurines are definitively absent (although lambeosaurines are known to have survived elsewhere^121^). Some of the remaining groups likewise appear to be locally extirpated from certain well-sampled localities (e.g., nodosaurids from the Hell Creek Formation, Montana; hadrosaurines from the Scollard Formation, Alberta) (Table 4), but this phenomenon may reflect habitat preferences or local sampling biases as opposed to genuine declines in numbers. The regional loss of species richness and beta diversity preceding the end-Cretaceous extinction is causally ambiguous, but plausibly attributed to the reestablishment of gene flow following the regression of the Western Interior Seaway the concomitant availability of larger habitats, and overall climate equability^137–139^. These larger habitats also fostered larger body sizes among herbivores (e.g., *Triceratops* spp., *Edmontosaurus annectens*, *Ankylosaurus magniventris*) and their predators (e.g., *Tyrannosaurus rex*). Body size correlates positively with dietary breadth among herbivores^5^, and thus the corresponding reduction in niche availability may have compounded the deleterious effects of increased gene flow on species diversity.

### Implications for the structuring of Late Cretaceous ecosystems

The DPF megaherbivore community appears to have been stable for the ~1.5 Myr deposition of the formation, evidenced by the continuous presence of ankylosaurids and nodosaurids (the latter are known from high in section, based on microsite material^140^), centrosaurines and chasmosaurines, and hadrosaurines and lambeosaurines. It was partly on this basis that Brinkman *et al*.^141^ and Mallon *et al*.^29^ described the fossil assemblage of the DPF as a chronofauna, defined as “a geographically restricted, natural assemblage of interacting animal populations that has maintained its basic structure over a geologically significant period of time”^142^. In fact, the same could be said for the entire Campanian-Maastrichtian vertebrate fossil assemblage of Laramidia^143^, which, in addition to the above taxa, supported the same predictable suite of fishes, lizards, snakes, turtles, champsosaurs, crocodilians, small ornithischians and theropods, and tyrannosaurids^12,135,144^. Olson^142^ linked chronofaunal persistence to stable environmental conditions, yet the Campanian-Maastrichtian of Laramidia was anything but stable, having witnessed extended periods of orogenic activity^138^, frequent forest fires^145^, and climatic fluctuations that resulted in changes to leaf physiognomy^89^ and reptile diversity^146,147^, among others. It may be that the ecological relationships examined here lent structure to the Late Cretaceous megaherbivore chronofauna, enabling its persistence^148^.

The regulating effects of competition on community structure may have further promoted faunal endemism during the Late Cretaceous (especially during the Campanian) of the North American western interior. Several authors^12,13,149^ have noted the existence of distinct faunal provinces across the ancient landscape of Laramidia (but see Lucas *et al*.^150^), but the associated diversity drivers remain elusive. Among those implicated are habitat fragmentation due to sea-level rise or orogeny, and floral zonation due to climatic gradients^12,135,138,149,151^. Longrich^152^ further argued for a role for competitive exclusion in explaining Laramidian provincialism, whereby species were prevented from immigrating into already established communities due to competition from the native fauna, leading to community isolation and increased beta diversity. He noted two predictions that follow from the competition hypothesis: (1) coordinated replacement in the fossil record, as older species are replaced by newer ones, and (2) geographic range expansion of species during times of reduced beta diversity (and thus, reduced competition). Prediction 1 is borne out by the present study, and is further supported by the demonstration of ecomorphological overlap between closely related species. Prediction 2 is likewise upheld by recent work, as discussed above. Thus, competition not only served to structure local megaherbivore communities, but also may have driven increases in beta diversity across the face of the Late Cretaceous western interior of North America.

### Megaherbivore ecology. 

Although both mammalian and dinosaurian megaherbivore communities appear to be shaped by bottom-up processes (i.e., limiting resources), this is hardly a foregone conclusion. Dinosaurs are not mammals, and the physiologies of the non-avian dinosaurs in particular likely ran the gamut from poikilothermy to homeothermy^153^. Thus, the mass-specific nutritional requirements of the megaherbivorous dinosaurs were, in all likelihood, less than those of their modern mammalian counterparts^52^. Further, the large body sizes of many of the carnivorous theropods, surpassing those of most modern carnivores (Fig. 1), means that even the megaherbivorous dinosaurs were not necessarily safe from top-down predation. However, the fact that predation did not entirely alleviate competitive interactions between the megaherbivores suggests that theropods may have favoured feeding on small or young individuals^11,67,154^. Finally, elevated atmospheric CO_2_ levels during the Late Cretaceous would have enhanced terrestrial primary productivity^155–158^, potentially reducing competitive strain among herbivores. In light of these considerations, the finding that both megaherbivorous mammals and dinosaurs were similarly resource-limited, despite their different evolutionary histories, physiologies, and exposure to otherwise very different conditions, is remarkable. It underscores the importance of resource availability in sustaining large herbivore diversity, independent of phylogeny.

## Conclusions

The model presented here is among the first to find quantitative support for competition-mediated megaherbivore community structure among dinosaurs. Like any model, it is only as good as the premises on which it is built, and its applicability both within and beyond the DPF is subject to testing with additional fossil discoveries. As the basic tenets of competitive exclusion are not in imminent danger of refutation, the model proposed here can be falsified by demonstrating (1) that megaherbivore species overlapped significantly in morphospace, and (2) that this overlap involved common taxa and persisted over time. Precisely what length of time is required to refute this model is difficult to say, but 300–600 Kyr seems reasonable, which is the average temporal range of a megaherbivore species within the DPF^29^. Where average species ranges are longer (e.g., 1.5–2 Myr in the Horseshoe Canyon Formation^130^), the amount of temporal overlap required to refute the proposed model would be correspondingly longer.

Further predictions might be teased from the present model. If megaherbivore niches were truly broader as a result of increased body size during the Maastrichtian (see Discussion), this might be reflected in the tooth wear or isotopic signals of *Ankylosaurus magniventris*, *Edmontosaurus annectens*, and *Triceratops* spp., particularly when compared to their Campanian predecessors. Exploratory work along these lines is certainly warranted.

Importantly, this study is based entirely on adult fossil material; immature individuals were excluded from this analysis (and those that preceded it) due to their rarity and incompleteness. Nonetheless, the young of megaherbivorous dinosaur species would have played an important role in their respective ecosystems, and may have been important competitors for the small herbivore species^159,160^. Follow-up studies on ontogenetic niche shifts in the megaherbivorous species, and their competitive likelihood at small body sizes, would further help to extend the present model.

It is worth stressing that this study does not attempt to explain *how* those ecomorphological differences arose among would-be competitors; it shows only *that* they arose, and explores the ecological implications that followed. Whether those differences between closely related (e.g., confamilial) taxa resulted from ecological displacement is worthy of investigation, but is beyond the scope of the present study. To this end, it would be interesting to investigate whether the feeding apparatuses of the clades examined here became more dissimilar to each other through their evolutionary histories, potentially reflecting character displacement due to ecological competition. It may be further possible to test whether more closely related (i.e., more ecomorphologically similar) taxa are more likely to go extinct as a result of sympatry, bolstering the character displacement hypothesis. Megaherbivore coexistence was evidently not a major evolutionary driver of cranial ornamentation during the Late Cretaceous^161^, but its role in promoting ecomorphological disparity via competition cannot yet be discounted.

## Supplementary information

Dataset S1

Dataset S2
